# Preoperative Prediction of Microsatellite Instability in Rectal Cancer Using Five Machine Learning Algorithms Based on Multiparametric MRI Radiomics

**DOI:** 10.3390/diagnostics13020269

**Published:** 2023-01-11

**Authors:** Yang Zhang, Jing Liu, Cuiyun Wu, Jiaxuan Peng, Yuguo Wei, Sijia Cui

**Affiliations:** 1 Cancer Center, Department of Radiology, Zhejiang Provincial People’s Hospital, Affiliated People’s Hospital, Hangzhou Medical College, Hangzhou 310014, China; 2 Medical College, Jinzhou Medical University, Jinzhou 121001, China; 3 Precision Health Institution, General Electric Healthcare, Hangzhou 310004, China

**Keywords:** rectal cancer, microsatellite instability, algorithm, radiomics

## Abstract

**Objectives:** To establish and verify radiomics models based on multiparametric MRI for preoperatively identifying the microsatellite instability (MSI) status of rectal cancer (RC) by comparing different machine learning algorithms. **Methods:** This retrospective study enrolled 383 (training set, 268; test set, 115) RC patients between January 2017 and June 2022. A total of 4148 radiomics features were extracted from multiparametric MRI, including T_2_-weighted imaging, T_1_-weighted imaging, apparent diffusion coefficient, and contrast-enhanced T_1_-weighted imaging. The analysis of variance, correlation test, univariate logistic analysis, and a gradient-boosting decision tree were used for the dimension reduction. Logistic regression, Bayes, support vector machine (SVM), K-nearest neighbor (KNN), and tree machine learning algorithms were used to build different radiomics models. The relative standard deviation (RSD) and bootstrap method were used to quantify the stability of these five algorithms. Then, predictive performances of different models were assessed using area under curves (AUCs). The performance of the best radiomics model was evaluated using calibration and discrimination. **Results:** Among these 383 patients, the prevalence of MSI was 14.62% (56/383). The RSD value of logistic regression algorithm was the lowest (4.64%), followed by Bayes (5.44%) and KNN (5.45%), which was significantly better than that of SVM (19.11%) and tree (11.94%) algorithms. The radiomics model based on logistic regression algorithm performed best, with AUCs of 0.827 and 0.739 in the training and test sets, respectively. **Conclusions:** We developed a radiomics model based on the logistic regression algorithm, which could potentially be used to facilitate the individualized prediction of MSI status in RC patients.

## 1. Introduction

Rectal cancer (RC) is one of the leading causes of cancer-related death worldwide, and it occurs with a series of genetic and protein abnormalities [1]. Of these, approximately 10% to 20% of RCs are caused by microsatellite instability (MSI), which manifests as the loss of one or more mismatch repair (MMR) proteins. In contrast, RC patients with microsatellite stability (MSS) have intact MMR proteins [2]. Research has shown that RC patients with MSI have unique biological behaviors and distinct responses to treatment, which may be resistant to 5-FU-based chemotherapy and more likely to benefit from immunotherapy [3,4,5]. Therefore, the MSI status of RC patients is a key predictor of treatment strategy and prognosis [6,7,8].

National Comprehensive Cancer Network (NCCN) and European Society for Medical Oncology (ESMO) guidelines both recommend detecting MSI status in RC patients [9,10]. It is worth noting that preoperative MSI assessment can only be performed by endoscopic biopsy [11,12]. However, the results of MSI detection may vary depending on insufficient samples or sampling techniques due to tumor heterogeneity [13,14]. The DNA extracted from the sample may not meet the minimum quality/quantity criteria for the genetic assay, thus resulting in unknown MSI status. In addition, the risks and complications of invasive biopsy limit its application in the real-time monitoring of disease progression and biological behaviors [15]. Therefore, it is valuable to develop a non-invasive, repeatable, and cost-effective MSI prediction method to guide clinicians to choose the next treatment strategy.

Radiomics can transform microscopic heterogeneity into quantitative features to capture the deep information of tumors [16,17,18]. A few scholars reported that radiomics based on enhanced CT have certain value in predicting MSI of colorectal cancer [19,20,21]. However, these studies were all based on CT and the subjects were colorectal cancer patients, while the incidence of MSI varies with the location of colorectal cancer [22,23,24]. In addition, two studies have found that CT-based tumor and peritumoral radiomic features can be used as important biomarkers for the preoperative prediction of MSI status [25,26]. With the development of imaging technology, MRI has gradually become the mainstream of preoperative tumor evaluation [27]. A recent study enrolled 199 RC patients found that the radiomics model based on multiparametric MRI have better predictive performance than those based on single unenhanced sequence images, with AUCs of 0.78 and 0.78 in the training and validation sets, respectively [28]. In addition, highly accurate and stable prediction model can be constructed by comparing different machine learning algorithms in order to improve the decision-making process in clinical practice [29]. Accordingly, the predictive value of MRI-based radiomics in evaluating the MSI of RC patients still deserves further attention.

Therefore, the purpose of this study was to extract radiomic features based on multiparametric MRI and construct a best noninvasive radiomic model by comparing different machine learning algorithms in order to better preoperatively predict the MSI status of RC patients. We believed that this predictive information will help stratify patients based on MSI status and help optimize decisions for personalized cancer treatment.

## 2. Materials and Methods

### 2.1. Patients

**Figure 1:**
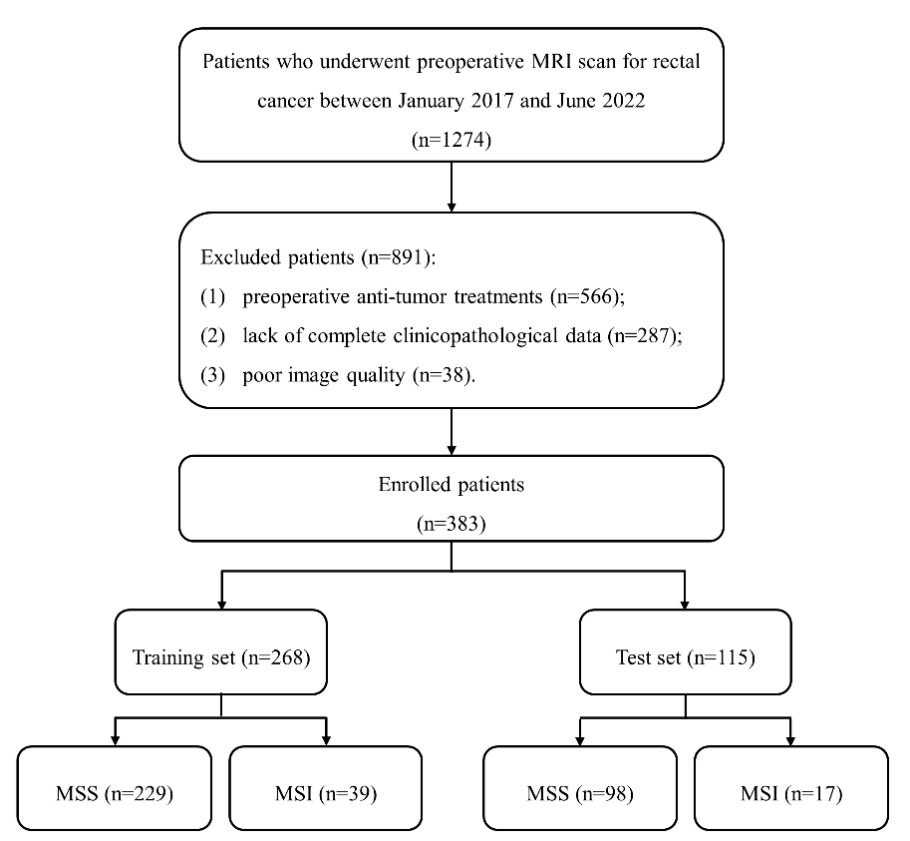
The patient recruitment process. (Note. MSS, microsatellite stability; MSI, microsatellite instability).

This retrospective study was approved by our institute review board, and written informed consent was waived. A total of 1274 patients with suspected RCs were included between January 2017 and June 2022. Inclusion criteria included: (1) pathologically proven RC; (2) received rectal MRI examinations one month before surgery; (3) no history of other malignant tumors. Exclusion criteria included: (1) preoperative anti-tumor treatments; (2) lack of complete clinicopathological data; (3) poor image quality caused by metal or motion artifact. Finally, 383 patients were enrolled and divided into training (n = 268) and test (n = 115) sets at a ratio of 7:3. The patient recruitment process is shown in Figure 1.

### 2.2. MRI Examinations

All MRI examinations were performed using a 3.0 T MRI scanner (Skyra; Siemens Healthineers, Erlangen, Germany) equipped with an 8-channel phased-array coil in supine position. The MRI protocol included the following sequences: (1) sagittal T_2_-weighted imaging (T_2_WI): repetition time (TR)/echo time (TE), 6060/90 msec; field of view (FOV), 180 × 180 mm^2^; matrix, 320 × 224; (2) axial T_2__blade_TSE: TR/TE, 4790/134 msec; FOV, 200 × 200 mm^2^; matrix, 384 × 451; (3) axial T_1_-weighted imaging (T_1_WI): TR/TE, 662/9.6 msec; FOV, 180 × 180 mm^2^; matrix, 320 × 224; (4) axial diffusion-weighted imaging (DWI) and apparent diffusion coefficient (ADC): TR/TE, 7330/56 msec; FOV, 200 × 200 mm^2^; matrix, 112 × 100; (5) contrast-enhanced T_1_WI (+C) was obtained by the intravenous injection of a gadolinium contrast agent (Magnevist, Bayer, Germany): TR/TE, 616/9.6 msec; FOV, 180 × 180 mm^2^; matrix, 320 × 224.

### 2.3. Clinical and Radiological Data

Clinical data from our picture archiving and communication system, including age, gender, carcinoembryonic antigen (CEA; levels greater than 5 ng/mL as abnormal), and carbohydrate antigens 19-9 (CA19-9; levels greater than 37 U/mL as abnormal), were retrospectively analyzed.

Radiological data were obtained from the structured report of rectum MRI, which included tumor size (maximum diameter of the tumor on the sagittal section), distance (DIS; distance from the end of the convex edge of the tumor to the edge of the anus), radiological tumor (T) stage, lymph node (N) stage, MRI-based extramural venous invasion (mrEMVI) status, circumferential resection margin (CRM), and anal canal invasion (ACI). These features were independently assessed by two experienced radiologists. For qualitative data, agreement was reached by negotiation when there was disagreement between the two radiologists. For quantitative data, measurements from these two radiologists were averaged.

### 2.4. Pathological Data

The MSI status of MMR proteins (MLH1, MSH2, PMS2, and MSH6) was evaluated by immunohistochemistry staining. RC patients were divided into MSI group and MSS group based on whether they were deficient in one or more MMR proteins [2]. Other pathological data included differentiation, pathological tumor (pT) stage, lymph node (pN) stage, and EMVI.

### 2.5. Tumor Segmentation

**Figure 2:**
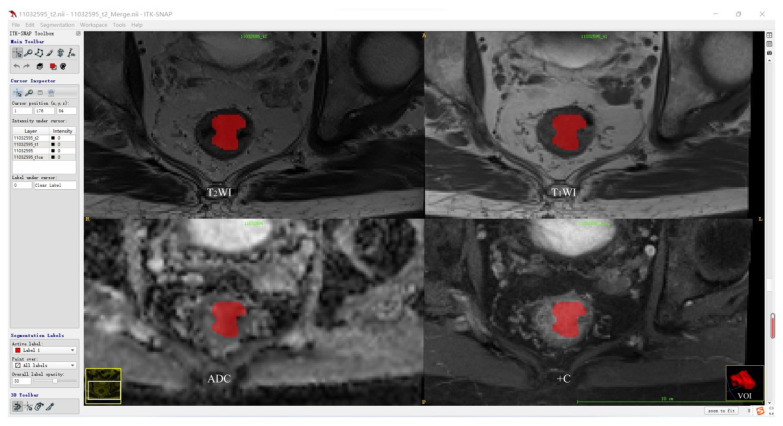
Tumor segmentation procedure.

Before tumor segmentation, A.K. software (Analysis Kit, GE Healthcare, Hangzhou, China) was used to adopt T_2_WI as the template for the rigid registration of T_1_WI, ADC, and +C sequences to ensure that the four sequences contained the same resolution, spacing, and origin. The standardized T_2_WI images were imported into open-source ITK-SNAP software, and the whole rectal tumor was segmented slice-by-slice to determine the volume of interest (VOI) for each patient by a radiologist with 5 years of experience in rectum MRI. According to the registration of different sequences, T_1_WI, ADC, and +C can share the same VOI obtained from T_2_WI. Then, the segmentation results were validated by another radiologist with more than 10 years of experience using intraclass correlation coefficient (ICC) on a cohort of 30 randomly selected patients. The tumor segmentation procedure is shown in Figure 2.

Figure 2 shows the representative results of the whole tumor on T_2_WI, T_1_WI, ADC, and +C sequences using ITK software. Three-dimensional volumetric reconstruction of segmented lesion is shown at the bottom right.

### 2.6. Radiomics Features Extraction and Selection

All segmented VOIs were imported into the Pyradiomics-based PHIgo software (GE Healthcare, V1.2.0, Hangzhou, China) for feature extraction. A total of 1037 radiomics features were extracted from each sequence, including four groups: (1) 18 first-order features; (2) 14 shape-based features; (3) 75 texture features: 16 gray level run length matrix features (GLRLM), 16 gray level size zone matrix (GLSZM), 5 neighboring gray tone difference matrix (NGTDM), 24 gray level co-occurence matrix features (GLCM), and 14 gray level dependence matrices (GLDMs); (4) 930 transform features: 186 Laplacian of Gaussian (LoG), and 744 wavelet transform features. T_2_WI, T_1_WI, ADC, and +C sequences were used, affording 4148 radiomics features per patient.

The ICCs of the measurements from the two radiologists were applied to evaluate inter-observer reliability and reproducibility. Features with ICCs > 0.80 were considered robust features. Then, dimension reduction was performed using analysis of variance, correlation test, univariate logistic analysis, and a gradient-boosting decision tree (GBDT) to reduce data redundancy and to further select the best significant radiomics features. Among them, gradient boosting sequentially combines weak learners in such a way that each new learner fits to the residuals from the previous step. The final features aggregate the results from each step and achieve powerful radiomics feature selection.

### 2.7. Model Construction and Evaluation

Five machine learning algorithms, including logistic regression, Bayes, support vector machine (SVM), K-nearest neighbor (KNN), and tree algorithms were used to construct radiomics models. The area under the receiver operating characteristic (ROC) curve (AUC) and DeLong test were used to evaluate the performance of different models. The 500 bootstrap method and its relative standard deviation (RSD) were taken to quantify the stability of these five algorithms. RSD = (the standard deviation of the 500 AUCs of each algorithm)/(the corresponding mean value of the 500 AUCs) × 100% [30]. The lowest RSD represented the best stability of the algorithm. Radiomics score (rad-score) was calculated via a linear combination of remaining features that were weighted by their respective coefficients to quantify the discriminability of the best radiomics model. The Hosmer–Lemeshow test were used to assess the goodness-of-fit of the best model. Then, patients were classified into high-risk and low-risk groups according to the best model to evaluate the predictive performance.

### 2.8. Statistical Analysis

Statistical analyses were performed with SPSS software (version 24.0, Chicago, IL, USA) and R software (version 3.4.1, Vienna, Austria). The two-sample t test or Mann–Whitney U test if not normally distributed was used for continuous variables, and results were expressed as mean ± standard deviation or median (interquartile range). Chi-squared test or Fisher’s exact test was used for categorical variables, and the results were expressed as numbers (percentages). Statistical significance was set at two-sided *p* < 0.05.

## 3. Results

### 3.1. Patients’ Characteristics

Among the 383 patients, the prevalence of MSI was 14.62% (56/383). In terms of MSS and MSI groups, there were no significant differences in any of the clinical, radiological, and pathological variables between the two groups in the whole, the training, and the test sets (*p* > 0.05, Table 1 and Table 2).

### 3.2. Radiomics Features Selection

**Figure 3:**
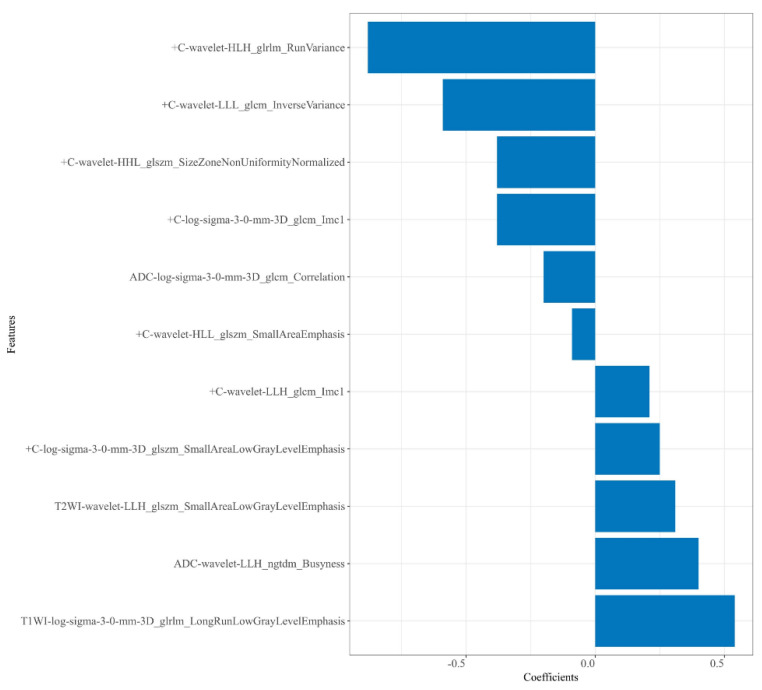
Plot of regression coefficients for retained radiomics features.

A total of 4148 radiomics features were extracted from each patient. Then, 2816 robust features with ICCs > 0.80 were obtained and used for dimension reduction. Firstly, analysis of variance on the 2816 robust features was performed. The variance of each feature was calculated, and then the features greater than the threshold 1 were retained. In this study, analysis of variance selected 1752 features. Secondly, dimensionality reduction of the selected 1752 features was performed using correlation test and univariate logistic analysis, and 234 and 22 features were selected successively. Following GBDT, 11 features were ultimately retained from the four sequences—T_2_WI (n = 1), T_1_WI (n = 1), ADC (n = 2), and + C (n = 7)—to construct the radiomics models. Among these 11 features, there were 4 LoG transform features and 7 wavelet transform features, as shown in Figure 3.

### 3.3. Model Construction and Comparison

**Figure 4:**
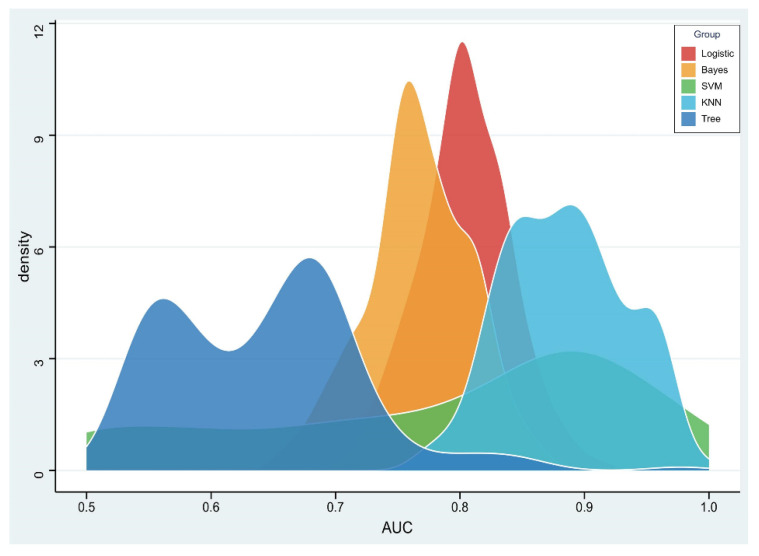
Density distribution of the area under the receiver operating characteristic (AUC) curve of radiomics constructed by five machine learning algorithms.

The RSD value of the radiomics model based on logistic regression was the lowest (4.64%), followed by Bayes (5.44%) and KNN (5.45%), which was significantly better than that of the SVM (19.11%) and tree (11.94%) algorithms, as shown in Figure 4.

**Figure 5:**
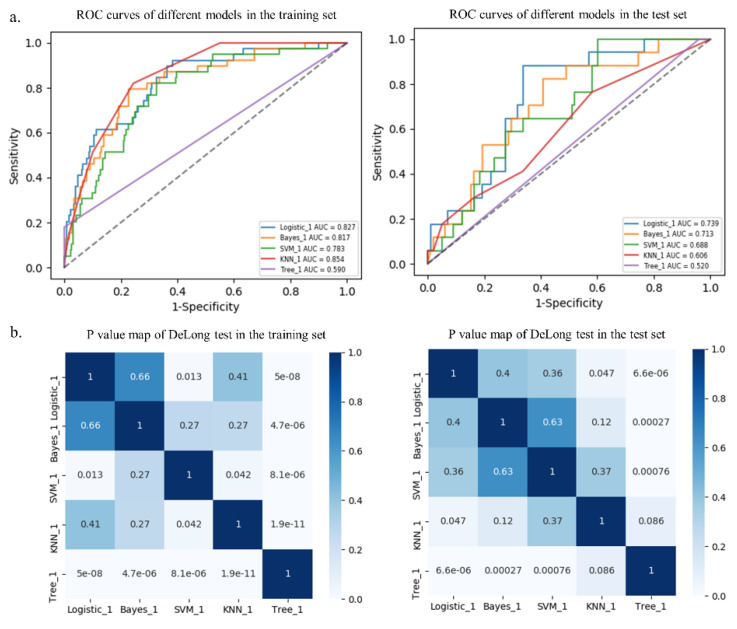
Receiver operating characteristic (ROC) curves of different models in the training and test sets (**a**). *p* value map of DeLong test in the training and test sets (**b**).

Among the five different radiomics models, the logistic model performed best with AUCs of 0.827 and 0.739 in the training and test sets, respectively, followed by the Bayes model with AUCs of 0.817 and 0.713, respectively, although there were no statistical differences (*p* > 0.05, Table 3 and Figure 5). In addition, the DeLong test showed that the prediction performance of the logistic model performed better than that of the SVM model (AUC = 0.783, *p* = 0.013) and the tree model (AUC = 0.590, *p* < 0.001) in the training set. Furthermore, the logistic model performed better than that of the KNN model (AUC = 0.606, *p* = 0.047) and the tree model (AUC = 0.520, *p* < 0.001) in the test set (Table 3 and Figure 5). Additional performance metrics of the logistic model (F1 score, 0.417; Matthews correlation coefficient, 0.337; G-mean, 0.723) were all higher than those of the other four models in the test set.

### 3.4. Logistic Model Verification

The logistic model exhibited good calibration in the training set (*p* = 0.401) and the test set (*p* = 0.153) using the Hosmer–Lemeshow test. The Rad-score was calculated using the following formula:

*Rad-score* = −2.467 + 0.542 × T_1_WI-log-sigma-3-0-mm-3D_glrlm_LongRunLowGrayLevelEmphasis

+ 0.305 × T_2_WI-wavelet-LLH_glszm_SmallAreaLowGrayLevelEmphasis

+ 0.403 × ADC-wavelet-LLH_ngtdm_Busyness

− 0.204 × ADC-log-sigma-3-0-mm-3D_glcm_Correlation

− 0.377 × +C-log-sigma-3-0-mm-3D_glcm_Imc1

+ 0.245 × +C-log-sigma-3-0-mm-3D_glszm_SmallAreaLowGrayLevelEmphasis

+ 0.210 × +C-wavelet-LLH_glcm_Imc1

− 0.087 × +C-wavelet-HLL_glszm_SmallAreaEmphasis

− 0.378 × +C-wavelet-HHL_glszm_SizeZoneNonUniformityNormalized

− 0.591 × +C-wavelet-LLL_glcm_InverseVariance

− 0.877 × +C-wavelet-HLH_glrlm_RunVariance

**Figure 6:**
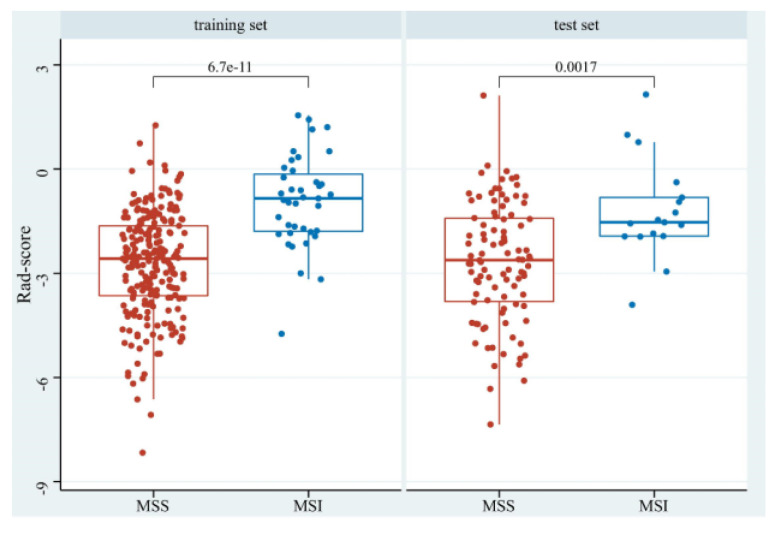
The boxplot of rad-scores of MSS and MSI in the training and test sets.

**Figure 7:**
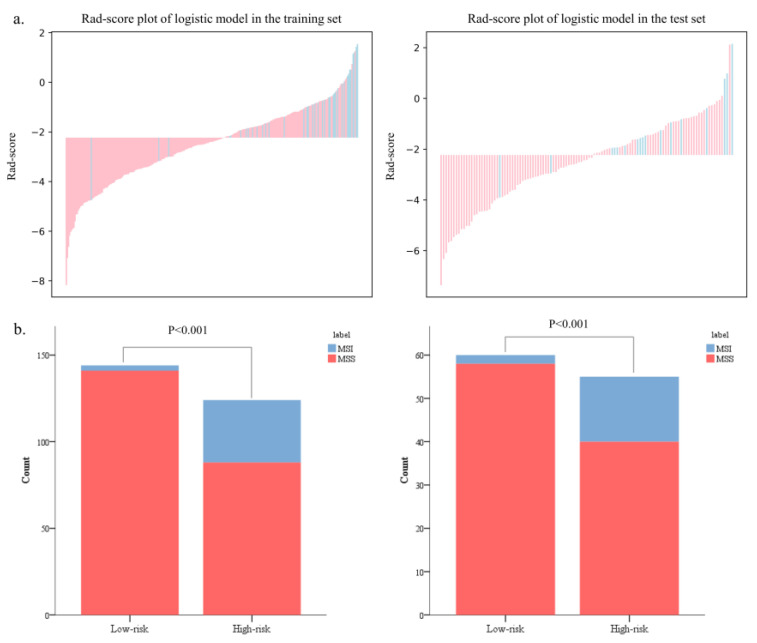
The bar charts of rad-scores in the training and test sets (**a**). The probability of MSI in the high-risk group was significantly higher than that in the low-risk group in both the training and test sets (**b**).

The Rad-scores in the MSI group were significantly higher than in the MSS group in both the training and test sets (*p* < 0.05, Figure 6). Red represents MSS, and blue represents MSI in the rad-score plot (Figure 7a). Patients with rad-scores greater than −2.260 were stratified into the high-risk group, and the others were stratified into the low-risk group. There were significant differences in the number of patients whose predicted MSI were between the low-risk and high-risk groups in both the training and test sets (*p* < 0.001), indicating the clinical applicability of the logistic model (Figure 7b).

## 4. Discussion

The preoperative prediction of MSI is of great significance for clinical decision making and prognosis. In this study, five different machine learning algorithms were compared, and the results showed that the logistic regression algorithm had the best stability. The logistic radiomics model based on multiparametric MRI can effectively predict MSI status and proved that it had great potential in the noninvasive preoperative prediction of MSI in RC patients.

Imaging can better capture the overall heterogeneity of the tumor and is superior to invasive tissue biopsy with sampling error due to insufficient samples or sampling techniques. Several studies have reported the correlation between CT-based radiomics and MSI status in colorectal cancer patients [19,20,21,22,23]. However, CT scan results in more radiation in patients receiving preoperative therapy and follow-up. Multiparametric MRI can provide more useful information and is recommended as the preferred examination for RC patients. Moreover, compared with MSS, colorectal cancer patients with MSI have distinct clinical and pathological features, including proximal colonic dominance and poor tumor differentiation [22,23]. In our study, the prevalence of MSI was 14.62% (56/383) in RC cases, which was consistent with the incidence of 10% to 20% in previous studies [26,31,32]. There were no significant differences in clinical, radiological, and pathological features between MSI and MSS in our study. Therefore, it is urgent to mine more in-depth quantitative radiomics features based on multiparametric MRI to predict MSI in RC patients.

Currently, only a few recently published studies have developed MRI-based radiomics for the preoperative prediction of MSI in RC patients [31,32,33]. However, the radiomics features extracted from these studies were all first-order, shape-based, and texture features. In addition to the above features, LoG and wavelet transform features were extracted in our study, in accordance with the Image Biomarker Standardization Initiative (IBSI) [34]. Surprisingly, the 11 features retained in our study were all LoG (n = 4) and wavelet (n = 7) transform features, indicating that transform features can capture more valuable information related to MSI in RC patients and better reflect the biological characteristics and heterogeneity of tumors [35]. Furthermore, the rad-score calculated based on these 11 features was significantly higher in MSI than in MSS, which was consistent with a previous study on CT-based radiomics for predicting MSI in colorectal cancer [22]. These findings indicate that quantitative radiomics features have certain value in predicting MSI in RC patients, which deserves further research and exploration.

In addition, radiomics models based on machine learning algorithms have attracted great attention to overfitting. Previous studies have used different algorithms to construct models [31,32,33]. The most valuable part of our study was the comparison of five different commonly used machine learning algorithms. The results showed that logistic regression algorithm had the lowest RSD (4.64%), followed by Bayes (5.44%) and KNN (5.45%), which was significantly better than that of SVM (19.11%) and tree (11.94%) algorithms. Therefore, the logistic regression algorithm with the best stability had great potential in predicting the MSI status of RC patients. Besides, the radiomics model based on the logistic regression algorithm performed best in predicting MSI, with AUCs of 0.827 and 0.739 in the training and test sets, respectively. Encouragingly, the logistic model can also afford the good classification and recognition of MSI status in RC patients, further demonstrating its superiority in clinical application. These confirmed that radiomics based on multiparametric MRI can noninvasively extract deeper quantitative image information and timely capture and reflect the biological characteristics of tumors. These also realized the preoperative individualized prediction of MSI status in RC patients, which was in line with the current trend of personalized and precise medicine.

Some limitations should be noted. Firstly, this retrospective study may lead to selection bias. Secondly, this study was a single-center study with a limited sample size. Therefore, further studies using large-scale multicenter prospective study are needed to reduce the impact of selection bias and to verify our findings. Finally, the manual segmentation may be affected by subjective evaluation, which may not be suitable for data processing in large samples. It is necessary to further find a suitable algorithm for automatic segmentation.

## 5. Conclusions

In conclusion, we compared and selected the optimal logistic regression machine learning algorithm to construct a radiomics model based on multiparametric MRI in this study. The logistic model was an effective and noninvasive approach for predicting the MSI status of RC patients and demonstrating better predictive performance, which could potentially be used to facilitate the individualized prediction of MSI status. Our study also provided important evidence for the potential use of the radiomics model for individualized treatment and improve the long-term survival outcomes of RC patients in the future.

## Figures and Tables

**Table 1:** Variables between MSS and MSI in the whole patients.

Variables	MSS (n = 327)	MSI (n = 56)	*p*
**Clinical features**			
Age, mean ± SD	64.5 ± 10.6	64.1 ± 10.1	0.784
Gender (men, %)	228 (69.7)	36 (64.3)	0.416
CEA (abnormal, %)	127 (38.8)	21 (37.5)	0.849
CA19-9 (abnormal, %)	31 (9.5)	6 (10.7)	0.773
**Radiological features**			
Size (IQR)	3.9 (1.8)	3.5 (2.0)	0.683
DIS (IQR)	7.7 (4.7)	7.9 (5.9)	0.850
T stage (T3–4, %)	235 (71.9)	44 (78.6)	0.297
N stage (positive, %)	200 (61.2)	39 (69.6)	0.226
mrEMVI (positive, %)	74 (22.6)	18 (32.1)	0.124
CRM (positive, %)	74 (22.6)	15 (26.8)	0.496
ACI (positive, %)	15 (4.6)	3 (5.4)	0.801
**Pathological features**			
Differentiation (n, %)			0.935
Poorly	56 (17.1)	10 (17.9)	
Moderately	249 (76.1)	43 (76.8)	
Well	22 (6.7)	3 (5.4)	
pT stage (T3–4, %)	218 (66.7)	38 (67.9)	0.880
pN stage (positive, %)	131 (40.1)	25 (44.6)	0.557
EMVI (positive, %)	112 (34.3)	20 (35.7)	0.879

**Table 2:** Variables of patients between MSS and MSI in the training and test sets.

Variables	Training Set (n = 268)			Test Set (n = 115)		
	MSS (n = 229)	MSI (n = 39)	*p*	MSS (n = 98)	MSI (n = 17)	*p*
**Clinical features**						
Age, mean ± SD	64.2 ± 10.4	64.3 ± 9.7	0.971	65.3 ± 11.0	63.8 ± 11.3	0.599
Gender (men, %)	158 (69.0)	27 (69.2)	0.977	70 (71.4)	9 (52.9)	0.129
CEA (abnormal, %)	93 (40.6)	14 (35.9)	0.578	34 (34.7)	7 (41.2)	0.606
CA19-9 (abnormal, %)	26 (11.4)	3 (7.7)	0.688	5 (5.1)	3 (17.6)	0.174
**Radiological features**						
Size (IQR)	3.8 (1.6)	3.5 (2.5)	0.632	4.0 (2.4)	3.8 (1.8)	0.956
DIS (IQR)	7.5 (5.0)	7.4 (5.1)	0.573	8.0 (4.1)	10.6 (6.8)	0.181
T stage (T3–4, %)	161 (70.3)	31 (79.5)	0.240	74 (75.5)	13 (76.5)	0.932
N stage (positive, %)	140 (61.1)	28 (71.8)	0.203	60 (61.2)	11 (64.7)	0.785
mrEMVI (positive, %)	48 (21.0)	12 (30.8)	0.174	26 (26.5)	6 (35.3)	0.652
CRM (positive, %)	55 (24.0)	11 (28.2)	0.575	19 (19.4)	4 (23.5)	0.948
ACI (positive, %)	10 (4.4)	3 (7.7)	0.624	5 (5.1)	0 (0.0)	0.758
**Pathological features**						
Differentiation (n, %)			0.954			0.528
Poorly	40 (17.5)	6 (15.4)		16 (16.3)	4 (23.5)	
Moderately	173 (75.5)	30 (76.9)		76 (77.6)	13 (76.5)	
Well	16 (7.0)	3 (7.7)		6 (6.1)	0 (0.0)	
pT stage (T3–4, %)	150 (65.5)	25 (64.1)	0.865	68 (69.4)	13 (76.5)	0.588
pN stage (positive, %)	97 (42.4)	19 (48.7)	0.488	34 (34.7)	6 (35.3)	0.962
EMVI (positive, %)	77 (33.6)	14 (35.9)	0.855	35 (35.7)	6 (35.3)	0.973

Note. MSS, microsatellite stability; MSI, microsatellite instability; CEA, carcinoembryonic antigen; CA19-9, carbohydrate antigens 19-9; DIS, distance from the end of the convex edge of the tumor to the edge of the anus; mrEMVI, MRI-based extramural venous invasion; CRM, circumferential resection margin; ACI, anal canal invasion; EMVI, extramural venous invasion; SD, standard deviation; IQR, interquartile range.

**Table 3:** Predictive performance of different models in training and test sets.

Models	Training Set			Test Set		
	AUC (95% CI)	Sensitivity	Specificity	AUC (95% CI)	Sensitivity	Specificity
Logistic	0.827 (0.776, 0.870)	0.923	0.616	0.739 (0.649, 0.816)	0.882	0.663
Bayes	0.817 (0.766, 0.862)	0.795	0.773	0.713 (0.622, 0.794)	0.824	0.592
SVM	0.783 (0.728, 0.830)	0.821	0.673	0.688 (0.595, 0.772)	1.000	0.398
KNN	0.854 (0.806, 0.894)	0.821	0.756	0.606 (0.510, 0.696)	0.765	0.418
Tree	0.590 (0.528, 0.649)	0.180	1.000	0.520 (0.425, 0.614)	1.000	0.041

Note. AUC, area under the receiver operating characteristic curve; CI, confidence interval.

## Data Availability

The data presented in this study are available on request from the corresponding author.
